# Phthalimide Analogs Enhance Genotoxicity of Cyclophosphamide and Inhibit Its Associated Hypoxia

**DOI:** 10.3389/fchem.2022.890675

**Published:** 2022-04-20

**Authors:** Amira M. Gamal-Eldeen, Hussein S. Agwa, Magdy A.-H. Zahran, Bassem M. Raafat, Sherien M. El-Daly, Hamsa J. Banjer, Mazen M. Almehmadi, Afaf Alharthi, Nahed M. Hawsawi, Fayez Althobaiti, Mona A. M. Abo-Zeid

**Affiliations:** ^1^ Clinical Laboratory Sciences Department, College of Applied Medical Sciences, Taif University, Taif, Saudi Arabia; ^2^ High Altitude Research Center, Prince Sultan Medical Complex, Taif University, Taif, Saudi Arabia; ^3^ Research & Development Department, Pharco B International Company for Pharmaceutical Industries, Borg El-Arab, Alexandria, Egypt; ^4^ Chemistry Department, Faculty of Science, Menoufiya University, Menoufiya, Egypt; ^5^ Radiological Sciences Department, College of Applied Medical Sciences, Taif University, Taif, Saudi Arabia; ^6^ Medical Biochemistry Department, National Research Centre, Cairo, Egypt; ^7^ Cancer Biology and Genetics Laboratory, Centre of Excellence for Advanced Sciences, National Research Centre, Cairo, Egypt; ^8^ Biotechnology Department, Faculty of Science, Taif University, Taif, Saudi Arabia; ^9^ Department of Cytology and Genetics, National Research Center, Cairo, Egypt

**Keywords:** hypoxia, cyclophosphamide, thalidomide, phthalimide, genotoxicity

## Abstract

Cyclophosphamide (CP) is a mutagen that is used in cancer chemotherapy, due to its genotoxicity and as an immunosuppressive agent**.** Thalidomide (TH) is another cancer chemotherapeutic drug. In this study, the cytogenotoxicity and hypoxia modulatory activities of two phthalimide analogs of TH have been evaluated with/without CP. Both analogs have increased CP-stimulated chromosomal aberrations than those induced by TH, including gaps, breaks/fragments, deletions, multiple aberrations, and tetraploidy. The analogs have elevated the cytotoxic effect of CP by inhibiting the mitotic activity, in which analog 2 showed higher mitosis inhibition. CP has induced binucleated and polynucleated bone marrow cells (BMCs), while micronuclei (MN) are absent. TH and analogs have elevated the CP-stimulated binucleated BMCs, while only analogs have increased the CP-induced polynucleated BMCs and inhibited the mononucleated BMCs. MN-BMCs were shown together with mononucleated, binucleated, and polynucleated cells in the CP group. Both analogs have elevated mononucleated and polynucleated MN-BMCs, whereas in presence of CP, TH and analogs have enhanced mononucleated and binucleated MN-BMCs. The analogs significantly induce DNA fragmentation in a comet assay, where analog 1 is the strongest inducer. The treatment of mice with CP has resulted in a high hypoxia status as indicated by high pimonidazole adducts and high HIF-1α and HIF-2α concentrations in lymphocytes. Analogs/CP-treated mice showed low pimonidazole adducts. Both analogs have inhibited HIF-1α concentration but not HIF-2α. Taken together, the study findings suggest that both analogs have a higher potential to induce CP-genotoxicity than TH and that both analogs inhibit CP-hypoxia via the HIF-1α-dependent mechanism, in which analog 1 is a more potent anti-hypoxic agent than analog 2. Analog 1 is suggested as an adjacent CP-complementary agent to induce CP-genotoxicity and to inhibit CP-associated hypoxia.

## Introduction

Cyclophosphamide (CP) is a synthetic alkylating cytostatic agent chemically related to the nitrogen mustards that is largely used as a chemotherapeutic agent in oncology and as an immunosuppressive agent (Ponticelli et al., 2018). The cytotoxic antitumor activities of CP are due to its journey in the liver, in which CP is converted into active metabolites aldophosphamide and phosphoramide mustards, which bind to DNA, thereby inhibiting DNA replication and initiating cell death, whereas another metabolic product, acrolein, elicits its noxious side effects (Kern and Kehrer, 2002). CP is not cell-cycle phase-specific, and its metabolites are capable of inhibiting protein synthesis through DNA and RNA crosslinking (Mills et a., 2019). Acrolein, phosphoramide mustard, and other free reactive species mediated by CP promote oxidative stress (Ponticelli et al., 2018).

According to the FDA, CP is primarily indicated to treat malignant lymphomas stages III and IV. These may comprise Hodgkin and non-Hodgkin lymphoma, lymphocytic lymphoma, small lymphocytic lymphoma, Burkitt lymphoma, and multiple myeloma, in addition to the treatment of breast cancer, ovarian adenocarcinomas, retinoblastoma, and disseminated neuroblastomas (Korkmaz et al., 2007; Mills et al., 2019). In addition to antimitotic and antineoplastic effects and as an active immunosuppressive agent with high selectivity for T cells, CP has been reported to be useful in the treatment of autoimmune diseases such as multiple sclerosis and as an immunosuppressant to prevent transplant rejection and graft–host complications (Emadi et al., 2009). A high CP dosage is used in eradication therapy of malignant hematopoietic cells, while lower dosages have exerted selective immunomodulation of regulatory T cells (Ahlmann and Hempel, 2016; Chatelanat et al., 2018).

**FIGURE 1:**
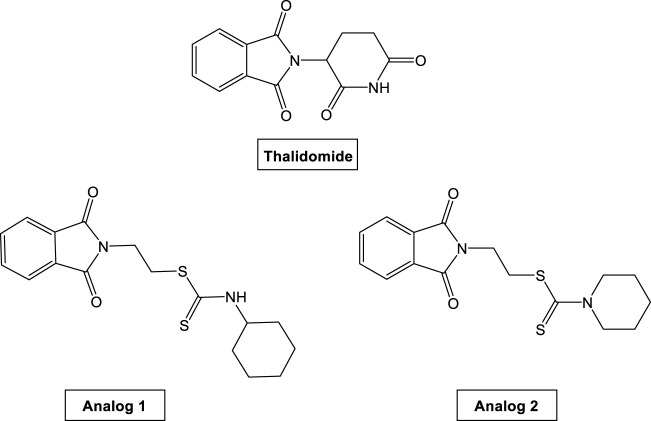
Chemical structures of TH and its analogs.

Thalidomide [TH (R,S)-2-(2,6-dioxo-3-piperidinyl)-1H-isoindole1,3(2H)-dione; Figure 1] was primarily administrated as an antiemetic agent and for the treatment of insomnia. In the last 2 decades, TH is used for the treatment of many hematological malignancies (Ribatti and Vacca, 2005) as well as variety of inflammatory and autoimmune diseases (Man et al., 2003) and in the treatment of multiple myeloma and other solid tumors (Juliusson et al., 2000). TH is yielding biologically active metabolites including phthalimide species that represents a pharmacophoric group as a suggested reason for their biological activities (Hashimoto, 2008).

Phthalimide is an imido derivative of phthalic acid. Phthalimides are hydrophobic and neutral and can therefore cross biological membranes *in vivo*. They are characterized by (–CO-N(R)-CO-) and an imide ring which support them to be biologically and pharmaceutically active (Kushwahaa and Kaushikb, 2016). Phthalimides have attracted attention due to their multiple functions as anti-inflammatory (Okunrobo et al., 2006), androgen receptor antagonists (Sharma et al., 2012), anticonvulsant (Kathuria and Pathak, 2012), antimicrobial (Khidre et al., 2011), anti-HIV-1 (Sharma et al., 2010), hypoglycemic (Mbarki and Elhallaoui, 2012), and anxiolytic (Yosuva and Sabastiyan, 2012) agents. There is an increased interest in the useful functions of phthalimides’ derivatives. Phthalimides have assisted as starting materials and intermediates for the synthesis of several alkaloids and pharmacophores (Kushwahaa and Kaushikb, 2016). Additionally, due to the evaluation of phthalimide derivatives as potential antitumor drug candidates (Chan et al., 2009; Sondhi et al., 2009) as well as the several biological activities of dithiocarbamates and their application in cancer treatment (Huang et al., 2009; Brahemi et al., 2010), our group had synthesized and reported that novel dithiocarbamate analogs are connected through either methylene or ethylene bridges to phthalimide pharmacophoric core (Zahran et al., 2017). These analogs, as shown in Figure 1, have shown remarkable antitumor activity and highly inhibitory activity on the vascular endothelial growth factor receptor (VEGFR). The introduction of an alkyl linker in between phthalimide and dithiocarbamate moieties in analogs 1 and 2 (Figure 1) added a value in improving their biological activities. Analogs 1 and 2 exhibited remarkable cytotoxicity against human breast MCF-7 adenocarcinoma cells and hepatocellular HepG2 carcinoma cells compared to TH. Both analogs have demonstrated high docking score values, and they significantly declined the concentration of VEGFR and consequently led to an inhibited tumor growth (Zahran et al., 2017). In continuation of this work for a better understanding of anti-cancer mechanism of these analogs, we designed the present study to investigate their cytogenotoxic and anti-hypoxic effect and consequently, their possible potentiation of CP genotoxicity.

## Experimental

### Synthesis of Analogs

Phthalimide dithioate derivatives were synthesized according to the procedure reported in our previous report (Zahran et al., 2017), which resulted in two TH analogs including analog **1** [2-(1, 3-Dioxoisoindolin-2-yl) ethyl cyclohexylcarbamodithioate] and analog **2** [2-(1, 3-Dioxoisoindolin-2-yl) ethyl piperidine-1-carbodithioate]. Their chemical structures are shown in Figure 1.

### Animal Model

Swiss albino mice (Age: 10 weeks; weight: 26 ± 1.8 g; sex: male) were obtained from the National Research Centre Animal Facility, Cairo, Egypt. The mice were divided into groups (*n* = 10), housed under standard conditions, and given standard food/water *ad libitum*. The mice experiments have been approved by the Ethical Committee Board. Tested analogs and CP were dissolved in sterile saline solution. The mice were injected intraperitoneally (IP) with 15 mg/kg body weight of the tested analogs. This dose proves safety in acute toxicity studies (data are not mentioned). Mice groups were injected with tested analogs, 4 h before IP-injection with CP (25 mg/kg body weight). After 1 day from treatment, blood and bone marrow specimens have been collected from mice. Control mice were administered saline by IP-injection, and CP-treated mice (CP-group) were administered IP-injected with CP alone (25 mg/kg body weight). To analyze chromosomal aberration, other groups under the same treatment settings were additionally IP-injected with colchicine (10 mg/kg body weight), 2 h before specimen collection.

### Chromosomal Aberrations

Chromosome aberrations analysis has been performed based on the method of Choudhury et al. (2000). Briefly, bone-marrow cells (BMCs) have been harvested from femurs in hypotonic solution. The cell suspension was incubated at 37°C for 20 min, before being centrifuged (1,000 rpm; 10 min). The cells were transferred to cold fixative (acetic acid: methanol; 1:3; V/V) and re-centrifuged before being submitted to chromosome staining and analysis. A hundred spread metaphases were examined per animal. Metaphases with gaps, numerical aberrations (polyploidy), fragments, chromatid breakage, and deletions have been noted.

### Mitotic Index

The same slides that have been stained for the analysis of chromosomal aberrations were utilized to analyze the mitotic index (MI), which depends on counting 1,000 cells per mouse. The number of dividing cells including prophases and metaphases was recorded. MI was calculated as the number of dividing BMCs/1,000 cells.

### Bone Marrow Micronucleus Assay

Both femurs of the mice were separated and the epiphyses were used to isolate BMCs (Valette et al., 2002). BMCs have been suspended in fetal calf serum and spread onto glass slides. BMC smears were set in six replicates for fixation (methanol; 10 min) and staining (May-Grunwald/Giemsa, pH 6). The number of nucleated BMCs and the micronucleated BMCs (MN-BMCs) have been scored according to the nuclei status (mono, bi, and poly) by scoring their count in 1,000 BMCs/mouse (D’Souza et al., 2002).

### Comet Assay

The comet assay was used to analyze the DNA damage in murine BMCs, based on the protocol of Collins (2004). The image analysis software (comet imager measurements software V2.2, Metasystems, Germany) connected to the fluorescent microscope (Zeiss, Germany; 40×) has been utilized to investigate and evaluate DNA damage/comet. A hundred BMCs/slide were examined. The quantification of tail moment (tail DNAl and mean migration distance of tail) has been performed in comparison to that of BMCs from control and CP-group using the nonparametric Mann–Whitney *U* test.

### Effect of Analogs on Hypoxia

The lymphocytes were isolated from the murine peripheral blood according to the method of Chi and Harris (1978). The lymphocytes have been stained with pimonidazole as an anoxic indicator (Challapalli et al., 2017), and then the cells were washed three times to remove excess pimonidazole. The cells have been digested using the cell lysis solution (#LSKCLS500; Merck, United States) supplemented with protease inhibitor cocktail (#P8340; Merck, United States), and the pimonidazole adducts were measured using a microplate fluorometer. In another experiment, the separated lymphocytes of different groups were lysed by the same buffer and the lysates were investigated for hypoxia-inducible factor-1 α (HIF-1α) and HIF-2α concentrations using the HIF-1α ELISA fluorescent kit (#ab229433; Abcam, Germany) and HIF-2-alpha ELISA kit (ab227898; Abcam, Germany).

### Statistical Analyses

For the statistical analysis of chromosome aberrations, Chi-square test (2X^2^ contingency table) was carried out. The Student’s *t*-test was used to analyze normally distributed data of mitotic index, micronucleus test, comet tail migration, and comet head diameter. ANOVA followed by a Tukey’s post hoc (at 99% confidence interval) was used to analyze HIF-1α and HIF-2α results. The differences between groups are considered significant at *p* < 0.05.

## Results and Discussion

The chromosomal instability underlying cancer pathogenesis has been lately approved as a fundamental event in the mitotic processes that disturbs the genome integrity (Ovejero et al., 2020). The development of the carcinogenesis process is usually correlated with the abnormalities of chromosomes that mainly result from frequent mutations in genes that contribute in preserving the stability of the chromosomes (Huang and Zhou, 2021). Those mutations evoke genomic instability events that can support tumor progression and further enhance metastasis (Lasolle et al., 2020). Additionally, other rearrangements and/or deletions have been detected in tumor suppressor genes, and the substitution single base has been detected in cancer cell clones with multiple mutations that can activate oncogenes (*e.g*., MYC and RAS) and inhibit tumor suppressor genes such as TP53 (Ma et al., 2021). Mitotic cells undergo DNA damage due to persisting interphase errors or due to the exposure to DNA damaging agents. Therefore, mitosis is a potential therapeutic target for a variety of chemotherapeutic drugs, including CP, that enhance prolonged mitotic arrest with the consequent accumulating DNA damage and cell death as the targeted final fate (Ovejero et al., 2020).

### Chromosomal Aberrations

In the current work, the administration of analogs alone has led to a significant disturbance in the chromosomal integrity in murine BMCs. The percentages of chromosomal gaps have been elevated significantly (*p* < 0.05) by both analogs. The percentages of total chromosomal aberrations, excluding gaps, have increased compared to control by analog 1 (*p* < 0.05) and analog 2 (*p* < 0.01). This significant induction is suggested to be due to a cumulative significant induction in fragment breaks, deletions, multiple aberrations, and tetraploidy, (Table 1), while TH treatment has resulted in no chromosomal aberrations. Additionally, the administration of CP alone has resulted in a high increase in the total chromosomal aberrations (*p* < 0.001) including fragment breaks (*p* < 0.05), deletions (*p* < 0.05), multiple aberrations (*p* < 0.001), and tetraploidy (*p* < 0.01), compared to the control (Table 1).

**TABLE 1:** Chromosomal aberrations and mitotic index analyses: BMCs from different mice groups have been investigated after 24 h from administration of TH and analogs in absence and presence of CP.

Groups	Different types of chromosomal aberrations										Total abnormal metaphases without gaps		Mitotic index
	Gap		Fragment or break		Deletion		Multiple aberration		Tetraploidy				
	No.	(%)	No.	(%)	No.	(%)	No.	(%)	No.	(%)	No.	%	No.
Control	7.0	1.6	13	2.7	1.1	0.5	n.d.	n.d.	2.4	0.2	18.0	3.6	32 ± 0.63
TH	10.1^a^ ^*^	2.1^a^ ^*^	18.1	3.3	n.d.	n.d.	n.d.	n.d.	3.2	0.2	21.3	3.5	22.16 ± 0.73^a^ ^*^
Analog 1	13.2^a^ ^**^	2.9^a^ ^**^	19.0^a^ ^*^	4.6^a^ ^*^	n.d.	n.d.	n.d.	n.d.	7.5^a^ ^**^	1.3^a^ ^**^	26.4^a^ ^*^	5.9^a^ ^*^	23.3 ± 2.08^a^ ^*^
Analog 2	12.2^a^ ^*^	3.1^a^ ^*^	23.3^a^ ^**^	5.4^a^ ^**^	5.3^a^ ^***^	0.9^a^ ^***^	3.0^a^ ^**^	0.4 ^a^ ^**^	8.1^a^ ^***^	1.9^a^ ^***^	39.6^a^ ^**^	8.8^a^ ^**^	25.4 ± 0.61^a^ ^*^
CP	22^a^ ^*^	5.2^a^ ^*^	33.1^a^ ^*^	6.6^a^ ^*^	3.0^a^ ^*^	1.2^a^ ^*^	91.5^a^ ^***^	21.5^a^ ^***^	14.1^a^ ^**^	2.8^a^ ^**^	158^a^ ^***^	32.0^a^ ^***^	19.0 ± 0.98^a^ ^**^
TH/CP	26.2^b^ ^*^	6.4^b^ ^*^	38.2^b^ ^*^	7.4^b^ ^*^	3.0	0.9	111.4^b^ ^*^	24.1^b^ ^*^	16.3	2.9	168.2	35.1^b^ ^*^	12.3 ± 1.16^b^ ^**^
Analog 1/CP	29.4^b^ ^*^	7.2^b^ ^*^	39.2^b^ ^***^	8.4^b^ ^***^	n.d.	n.d.	119.2^b^ ^*^	28.73^b^ ^*^	19.6^b^ ^*^	2.9	177.8^b^ ^*^	39.6^b^	9.1 ± 0.97^b^ ^***^
Analog 2/CP	30.4^b^ ^**^	7.7^b^ ^**^	46.2^b^ ^**^	9.1^b^ ^**^	5.0^b^ ^**^	0.9^b^ ^**^	132.1^b^ ^**^	31.4^b^ ^**^	24.7^b^ ^**^	2.7	197.9^b^ ^**^	46.1^b^ ^***^	8.8 ± 0.72^b^ ^***^

The number of investigated BMCs was 500 cells (*n* = 10). Mice were treated with TH, analog 1, and analog 2 (15 mg/kg body wt.) or CP (25 mg/kg body wt.).

*p < 0.05; **p < 0.01; ***p < 0.001.

a Compared to the control group.

b Compared to the CP-group. Aberrations were analyzed by X^2^ tests, and the mitotic index was analyzed by the *t*-test.

The administration of TH, analog 1, or analog 2 to mice before CP resulted in a dramatic induction in the aberration percentages, without gaps, compared to the CP-group. This induction (*p* < 0.001) of the total chromosomal aberrations has been detected as 168.2, 177.8, and 197.9% in TH/CP, analog 1/CP ,and analog 2/CP groups, respectively (Table 1). It is clear that analog 1 and analog 2 strongly induced the same aberrations types of CP-like gaps, fragments, breaks, deletions, multiple aberrations, and tetraploidy (*p* < 0.0*5–p* < 0.001). Conclusively, both analogs 1 and 2 enhance the genotoxicity of CP.

### Mitotic Index

The mitotic index has been dramatically diminished in the CP-group (*p* < 0.01) compared to the control. The BMC proliferation rate has been depressed (*p* < 0.05) in analog 1 and analog 2 treated groups, compared to the control mice, (Table 1). The rate was remarkably inhibited in TH/CP (*p* < 0.01), analog 1/CP (*p* < 0.001) and analog 2/CP groups (*p* < 0.001), compared to the CP-group, as shown in Table 1. These results suggest that the analogs, to variable degrees, induced CP cytotoxicity and inhibited the mitosis, and that TH exhibited the lowest mitosis suppression, while analog 1 and analog 2 possessed potential inhibitory affinity for the mitotic activity (Table 1).

### Bone Marrow Micronuclei

The MN assay affords a sensitive recognition of structural chromosomal damages in BMCs (Fenech, 2000). MN are minor chromatin bodies, which occur after the fragment condensation of acentric chromosome in the cytoplasm, usually encouraged by clastogenics or spindle-poison in dividing cells (Fenech, 2000). MN incidences have been recognized as a consistent index for chromosome loss and breaks (Lajmanovich et al., 2005).

Table 2 demonstrates that the nuclear impairment was remarkable in the CP-group. The total MN-BMC number has increased significantly (*p* < 0.001), when the animals were treated with TH, analog 1, and analog 2. MN-BMCs existed together with the variable types of BMCs including mononucleated, binucleated, and polynucleated cells. All of the three types of MN-BMCs have elevated dramatically (*p* < 0.001) in the CP-group, compared to the control mice. The treatment with TH/Cp, analog 1/Cp, and analog 2/Cp has remarkably increased this mononucleated MN-BMC induction (*p* < 0.05, *p* < 0.05 and *p* < 0.001, respectively). Likewise, binucleated MN-BMCs have elevated significantly (*p* < 0.05, *p* < 0.05 and *p* < 0.01, respectively), while analog 1/Cp and analog 2/Cp showed a remarkable inhibition (*p* < 0.001) in polynucleated MN-BMCs compared to the CP-group.

**TABLE 2:** Micronuclei analysis: BMCs from different mice groups have been investigated after 24 h from administration of TH and analogs in absence and presence of CP.

Group	Total MN-BMCs per 1000 BMCs (mean ± SE)	BMCs with MN per 500 BMCs (mean ± SE)		
		Mononucleated BMC	Binucleated BMC	Polynucleated BMC
Control	7.51 ± 0.92	4.82 ± 0.81	0.54 ± 0.14	0.11 ± 0.10
TH	28.7^a^ ^***^ ± 2.36	9.67^a^ ^*^ ± 1.73	0.80 ± 0.12	n.d.
Analog 1	37.13^a^ ^***^ ± 2.55	18.44^a^ ^***^ ± 2.54	0.98 ± 0.28	0.51^a^ ^***^ ± 0.16
Analog 2	41.29^a^ ^***^ ± 3.43	16.89^a^ ^***^ ± 3.45	1.42^a^ ^*^ ± 0.22	0.43^a^ ^***^ ± 0.18
CP	51.10^a^ ^***^ ± 3.95	31.88^a^ ^***^ ± 2.25	6.01^a^ ^***^ ± 0.93	6.61^a^ ^***^ ± 2.04
TH/CP	66.20^b^ ^*^ ± 4.87	40.64^b^ ^*^ ± 3.26	9.26^b^ ^*^ ± 2.13	6.96 ± 0.82
Analog 1/CP	82.32^b^ ^**^ ± 5.33	49.94^b^ ^*^ ± 3.43	10.66^b^ ^*^ ± 1.93	0.71 ± 0.16^b^ ^***^
Analog 2/CP	78.74^b^ ^**^ ± 5.66	52.23^b^ ^***^ ± 6.78	16.12^b^ ^***^ ± 2.17	0.78 ± 0.37^b^ ^***^

The total number of scored BMCs is 1,000 BMCs/mouse, (*n* = 10 mice/group).

*p< 0.05; **p< 0.01; ***p< 0.001.

a Compared to the control group.

b Compared to the CP-group (*t*-test).

Moreover, the intact BMC type (mononucleated cells), plus binucleated and polynucleated types were detected without MN (Table 3). In the CP-group, binucleated and polynucleated BMCs, without MN, showed a noticeable induction (*p* < 0.001), while the regular mononucleated cells showed a remarkable inhibition (*p* < 0.05), compared to the control. Mononucleated BMCs, without MN, have significantly declined (*p* < 0.05) in analogs groups. The binucleated BMCs, without MN, have noticeably elevated in TH/CP (*p* < 0.05), analog 1/CP (*p* < 0.05), and analog 2/CP (*p* < 0.01) groups. Polynucleated BMCs are elevated in the analog 2/CP group, compared to the CP-group. The analogs have enhanced the tetraploid BMC number in the chromosomal aberration analysis and binucleated and polynucleated BMC numbers in CP-stimulated with MN, which suggests that diploid cells with aberrations have impaired cell division as a consequence of interstrand crosslinks (Krishnaswamy and Dewey, 1993; Martínez et al., 2005). These findings indicate the mutagenic efficacy of the analogs.

**TABLE 3:** Analysis of multinucleated BMCs in absence of micronuclei: BMCs from different mice groups have been investigated after 24 h from administration of TH and analogs in absence and presence of CP.

Group	BMCs without micronuclei (mean ± SE)		
	Mononucleated BMC	Binucleated BMC	Polynucleated BMC
Control	990.00 ± 13.35	9.41 ± 0.71	1.43 ± 0.62
TH	918.11 ± 9.76	16.75^a^ ^*^ ± 0.98	2.76 ± 0.27
Analog 1	907.24^a^ ^*^ ± 8.65	19.83^a^ ^*^ ± 0.85	2.90^a^ ^*^ ± 0.65
Analog 2	894.38^a^ ^*^ ± 13.72	17.12^a^ ^*^ ± 0.69	4.12^a^ ^***^ ± 0.97
CP	887.84^a^ ^*^ ± 10.95	42.71^a^ ^***^ ± 10.25	7.6^***^ ± 1.55
TH/CP	874.4 ± 9.27	53.44^b^ ^*^ ± 9.88	7.78 ± 0.76
Analog 1/CP	852.89^b^ ^*^ ± 12.72	64.78^b^ ^*^ ± 10.33	9.76 ± 1.93
Analog 2/CP	836.34^b^ ^*^ ± 12.76	66.15^b^ ^**^ ± 8.77	11.91^b^ ^*^ ± 1.97

The total number of scored BMCs is 1,000 BMCs/mouse (*n* = 10 mice/group).

*p< 0.05; **p< 0.01; ***p< 0.001.

a Compared to the control group.

b Compared to the CP-group (*t*-test).

The comet assay provides a tool to detect DNA damage and multiple DNA impairment types (e.g., single and double-strand breaks, alkali-labile sites, incomplete repair loci, cross-links, and fragmentation) (Azqueta et al., 2020). In the current study, a dramatic induction in DNA damage (*p* < 0.001) was observed in the CP-group as designated by the comet tail length/tail moment compared to their corresponding control (Table 4). Mice treated with analogs alone showed dramatic changes in the comet pattern. The findings demonstrated that the analogs are strong inducers of the pre-induced-DNA damage that lead to elevated tail lengths and tail moments. Analog 1 possesses the highest damaging affinity to DNA, as shown in Table 4. However, in analogs/CP-group, the results indicated that the analogs stimulate a further elevation of the CP-induced DNA damage that leads to the stimulation of the pre-induced-DNA damage, as concluded from the elevated tail lengths and moments (Table 4).

**TABLE 4:** Analysis of DNA damage and hypoxia indicators: DNA damage has been analyzed by comet assay in BMCs, while hypoxia has been monitored in lymphocytes by the determination of total hypoxia/pimonidazole adducts, HIF-1α, and HIF-2α in lymphocytes. Cells from different mice groups have been investigated after 24 h from administration of TH and analogs in absence and presence of CP. The data are represented as mean ± S.E.

Treatment	DNA damage (*n* = 10)		Hypoxia indicator		
	Tail moment	Tail length (µm)	Pimonidazole adduct (RFU)	HIF-1α (ng/ml)	HIF-2α (ng/ml)
Control	0.38 ± 0.41	23.33 ± 1.71	921 ± 98	31.12 ± 3.62	66.12 ± 7.01
TH	2.34 ± 0.91	38.41 ± 3.09^a^ ^*^	1,188 ± 19	41.42 ± 5.05	71.33 ± 8.77
Analog 1	2.96 ± 0.84	67.74 ± 3.76^a^ ^***^	973 ± 10	24.24 ± 2.73	56.18 ± 7.32
Analog 2	3.98 ± 0.95	54.17 ± 4.43^a^ ^***^	1,207 ± 14	34.23 ± 2.54	58.66 ± 7.93
CP	8.11 ± 1.23	71.04 ± 8.02^a^ ^***^	3,253 ± 39^*^ ^b^	408.51 ± 46.31^a^ ^***^	643.13 ± 72.34^a^ ^***^
TH/CP	12.51 ± 2.29	83.21 ± 6.66^b^ ^**^	3,441 ± 22	431.91 ± 51.74	661.08 ± 80.12
Analog 1/CP	13.76 ± 2.16	99.18 ± 6.71^b^ ^**^	1,293 ± 81^***^ ^b^	223.24 ± 25.02^b^ ^**^	548.93 ± 63.84
Analog 2/CP	15.74 ± 2.67	91.88 ± 7.08^b^ ^**^	1,409 ± 19^***c^	283.35 ± 32.42^b^ ^*^	580.22 ± 81.24

*p< 0.05; **p< 0.01; ***p< 0.001.

a Compared to the control group.

b Compared to the CP-group (*t*-test).

The previous report suggests that low CP dose interacts with the variable types of tumor microenvironment (TME) cells, including tumor-infiltrating lymphoid cells, endothelial cells, pericytes, and recruited circulating endothelial cells (Picoli et al., 2021). The anti-angiogenic mechanisms produced by CP have been identified by previous experimental studies, resulted in a declined microvessel density and then the enhancement of hypoxia (Shahrzad et al., 2008; Laheurte et al., 2020). The pathways of hypoxic stress response, largely controlled by HIF, are highly involved in the regulation of immune cells function, as a major regulator of immune cell metabolic function. HIF expression and stabilization in immune cells can be triggered by hypoxia among other factors (Chen and Gaber, 2021). Hypoxia status in TME is firmly associated with increased resistance to chemotherapy. Hypoxia induces HIFs that upregulate the expression of many drug-resistant-related genes that ultimately led to tumor resistance to chemotherapy (McAleese et al., 2021). The HIF function is determined by HIF-1a and HIF-2a (Hsu et al., 2020). Therefore, we studied the CP influence on hypoxia status in lymphocytes. In the current study, the treatment with CP resulted in a high hypoxia status as indicated by the formation of pimonidazole adducts and the remarkable induction of HIF-1α and HIF-2α concentrations (*p* < 0.001), in mice lymphocytes (Table 4). On the other hand, TH and the analogs showed a non-significant change in hypoxia degree compared to control mice, while in analogs/CP-treated mice, the results revealed that only the analogs have inhibited the CP-induced-hypoxia as concluded from the low pimonidazole adducts (*p* < 0.001), (Table 4). The further investigation of the analogs’ effect on HIF-1α and HIF-2α concentrations indicated that both analogs have a non-significant effect on HIF-2α, on contrary both analog 1 and analog 2 significantly have inhibited HIF-1α concentration (*p* < 0.01 and *p* < 0.05, respectively), (Table 4). These findings suggest that inhibition of hypoxia by both analogs is HIF-1α-dependent and that analog 1 is a more potent anti-hypoxic agent.

Several studies had shown that CP exposure enhances intracellular reactive oxygen species (ROS) generation, which consumes the cellular oxygen (Manda and Bhatia, 2003; Emmenegger et al., 2006). CP had been reported to significantly increase HIF-1α protein levels, which was accompanied by an induction in macrophage infiltration and elevated iNOS levels (Viola et al., 2008). These facts are linked to that after radiation treatment whereby nitric oxide induction was responsible for aerobic stabilization of HIF-1α via nitrosylation of a cysteine residue in the oxygen-dependent degradation domain, which prohibited the recognition of the protein by the von Hippel–Lindau complex (Li et al., 2007). As reviewed in Penel et al. (2012), multiple previous studies have reported that the combination of CP with other effective agents markedly enhanced the CP anti-cancer effect through multiple mechanisms in these tumor-bearing mice models including classic cytotoxic drugs (paclitaxel, doxorubicin, and cisplatin), immunostimulant agents (thalidomide (Zhao et al., 2005), lenalidomide, and interleukin-2), molecule-targeting hypoxic cells (tirapazime and hyperthermia) (Emmenegger et al., 2006), and molecular-targeting agents (sunitinib, imatinib, and trastuzumab). Similarly, the current study is an in-line trial to investigate the derivatives of phthalimide (as effective part of TH), which are dithiocarbamate analogs connected through either methylene or ethylene bridges to phthalimide pharmacophoric core, as cytogenotoxic and anti-hypoxic agents to potentiate CP anti-cancer activity. The study findings suggest that both analogs have a high potential to increase CP-genotoxicity and to inhibit CP-hypoxia via the HIF-1α-dependent mechanism. Analog 1 is a potent anti-hypoxic agent, and it is suggested as a promising adjacent CP-complementary agent to induce CP-genotoxicity and to suppress CP-associated hypoxia.

Taken together, the study findings suggest that both analogs have a higher potential to induce CP-genotoxicity than TH and that both analogs inhibit the CP-hypoxia via HIF-1α-dependent mechanism, in which analog 1 is a more potent anti-hypoxic agent than analog 2. Analog 1 is suggested as an adjacent CP-complementary agent to induce CP-genotoxicity and to inhibit CP-associated hypoxia. In conclusion, both analogs have increased CP-stimulated chromosomal aberrations than those induced by TH. The analogs have elevated the cytotoxic effect of CP by inhibiting the mitotic activity, in which analog 2 showed higher inhibition. In the absence of MN, CP has induced bi- and poly-nucleated BMCs. TH and analogs have elevated the CP-stimulated bi-nucleated BMCs, while only analogs have increased the CP-induced poly-nucleated BMCs and inhibited the mono-nucleated BMCs. CP-induced MN-BMCs were accompanied with mono-, bi-, and poly-nucleated cells. Both analogs remarkably have elevated mono- and poly-nucleated MN-BMCs. In the presence of CP, TH and analogs have enhanced mono- and bi-nucleated MN-BMCs. The analogs significantly induce DNA fragmentation, in which analog 1 is the strongest inducer. CP treatment has resulted in a high hypoxia status as indicated by high pimonidazole adducts and HIF-1α/HIF-2α concentrations in lymphocytes. Analogs/CP-treated mice showed low pimonidazole adducts. Both analogs have inhibited HIF-1α concentration but not HIF-2α. Comparing the results of both analogs indicated that analog 2 was a higher inducer of chromosomal aberrations and inhibitor of mitosis than analog 1. On the other hand, analog 1 was a higher inducer of DNA-damage and MN formation and a higher inhibitor of hypoxia than analog 2.

## Data Availability

The raw data supporting the conclusion of this article are available within the article.
